# Pathological Characteristics of Aspirated Thrombi From Coronary Artery Embolism in Patients With Acute Myocardial Infarction

**DOI:** 10.7759/cureus.96443

**Published:** 2025-11-09

**Authors:** Takao Konishi, Naohiro Funayama, Daisuke Sunaga, Katsuhiko Ohori, Tadashi Yamamoto, Yusuke Kashiwagi, Daisuke Hotta, Kenji Yamazaki, Hiroshi Nishihara, Shinya Tanaka

**Affiliations:** 1 Department of Cardiology, National Defense Medical College, Tokorozawa, JPN; 2 Advanced Medical Research Institute, Hokkaido Cardiovascular Hospital, Sapporo, JPN; 3 Department of Cardiology, Hokkaido Cardiovascular Hospital, Sapporo, JPN; 4 Center for Cancer Genomics, Keio University School of Medicine, Tokyo, JPN; 5 Department of Cancer Pathology, Hokkaido University, Sapporo, JPN

**Keywords:** acute myocardial infarction, aspiration thrombectomy, coronary artery embolism, pathology, percutaneous coronary intervention

## Abstract

Background: The pathological differences in aspirated thrombi between atherosclerotic causes and coronary artery embolism (CE) in patients with acute myocardial infarction (AMI) remain poorly understood. The purpose of this study was to compare the pathological features of aspirated thrombi between AMI patients with CE and those with atherosclerotic causes (non-CE).

Methods: We analyzed coronary thrombi retrieved between 2015 and 2019 from 89 consecutive patients presenting with de novo AMI, of whom four had CE and 85 did not. CE was diagnosed based on angiographic and other diagnostic imaging findings. The samples were stained with hematoxylin and eosin (H&E) and elastica-Masson stain (EM). Immunohistochemistry was performed using specific antibodies against CD34 and CD68 to detect endothelial cells and macrophages, respectively. The aspirated thrombi were assessed for their histopathological characteristics.

Results: Atrial fibrillation (AF) was more frequently documented in patients with CE than in those with non-CE (100% vs. 9%). The erythrocytes and erythrocytes/total thrombus areas were greater. The prevalence of erythrocyte-dominant thrombi was significantly higher in patients with CE than in those with non-CE (4.94 mm² (3.38-7.35 mm²) vs. 0.62 mm² (0.05-2.51 mm²); 55.6% (50.9-57.7%) vs. 12.6% (2.0-36.2%), P = 0.002; and 75% vs. 7%, P = 0.003, respectively). The age of aspirated thrombi was significantly fresher in CE than in the non-CE (“fresh”: 75% vs. 18%, “lytic”: 25% vs. 28%, and “old”: 0% vs. 54%, respectively).

Conclusions: This study suggests that aspirated thrombi derived from patients with AMI due to CE are more erythrocyte-dominant and fresher than those derived from patients with AMI due to atherosclerotic causes. Identifying the etiology of patients with AMI is important because anticoagulation therapy is essential for secondary prevention in patients with CE.

## Introduction

Although plaque rupture, plaque erosion, and calcified nodules are the most frequent causes of acute myocardial infarction (AMI) [1,2], coronary artery embolism (CE) is also an important nonatherosclerotic etiology. The prevalence of CE in patients with AMI has been reported to be 3%-7% [3,4]. However, current antiplatelet therapy without anticoagulants may cause coronary thromboembolic events, which tend to be recurrent, multiple, and unpredictable in patients with CE. Despite advancements in techniques and devices, recent studies reported a one-year occurrence of major adverse cardiac events (MACEs) of 3.5%-8.7% following percutaneous coronary intervention (PCI) in patients with acute coronary syndrome [5,6]. Accurate diagnosis of CE can contribute to optimal secondary prevention, potentially reducing the rate of MACEs after PCI in patients with AMI.

Previous studies have shown that aspiration thrombectomy in patients with a high thrombus burden can decrease thrombus volume, reduce the occurrence of distal embolization, improve thrombolysis in myocardial infarction (TIMI) grade flow, ameliorate microvascular perfusion, and consequently improve clinical outcomes [7,8]. However, routine aspiration thrombectomy during primary PCI is not recommended by the current guideline (class III) [9,10]. In contrast, many pathological studies on aspirated thrombi derived from patients with AMI have been conducted to investigate the relationship between thrombus components and AMI prognosis and to identify the underlying etiology of acute coronary thrombosis [11-18]. In 164 patients with ST-elevation myocardial infarction (STEMI), a previous pathological study showed that erythrocyte area in the thrombi and other clinical characteristics, including glucose levels on admission, larger vessel diameter (≥ 3.5 mm), and pre-balloon dilation, were independent predictors of angiographically visible distal embolization [16]. Another study demonstrated that organized thrombus, age, history of AMI, and the presence of shock were independently associated with in-hospital death (P < 0.05) [15]. However, the pathological characteristics of aspirated thrombi in patients with AMI due to CE have not been fully investigated.

Therefore, this study aimed to identify the pathological characteristics of aspirated thrombi from CE and compare them with those from atherosclerotic causes in patients with AMI who underwent aspiration thrombectomy during PCI.

## Materials and methods

Sample population

We retrospectively analyzed all the patients with AMI referred to the catheterization laboratory for primary PCI associated with aspiration thrombectomy performed using a low-profile catheter (Export Advance 6Fr or 7Fr, Medtronic, Santa Rosa, CA) at Hokkaido Cardiovascular Hospital, Sapporo, Japan, between July 2015 and February 2019 (n = 317). Aspiration thrombectomy was performed whenever possible in all patients with a TIMI flow grade of 0 and all patients with a visible thrombus if the TIMI flow grade was ≥1. Two hundred eighteen patients were excluded because they had not undergone aspiration thrombectomy during PCI, or the thrombus material obtained by aspiration thrombectomy was insufficient for pathological analysis, and 10 patients had developed stent thrombosis. Ultimately, 89 patients (71 men and 18 women) were enrolled in the study. 

Written informed consent was obtained from patients or their relatives before PCI (approval number: 2018). The Ethics Committee of Hokkaido Cardiovascular Hospital approved the study. This study was conducted in compliance with the Declaration of Helsinki and ethical principles for medical research involving human participants.

Definitions

Diabetes mellitus was defined as (a) plasma glucose of ≥ 126 mg/dl in the fasting state, or (b) ≥ 200 mg/dl, two hours after an oral glucose load, or (c) ≥ 6.5% plasma HbA1c, or (d) use of insulin or an oral hypoglycemic medication. Hypertension was defined as ≥ 140 mmHg systolic blood pressure, ≥ 90 mmHg diastolic blood pressure, or use of an antihypertensive medication. Dyslipidemia was defined as ≥ 220 mg/dl total cholesterol, ≥ 140 mg/dl low-density lipoprotein cholesterol, ≤ 40 mg/dl high-density lipoprotein cholesterol, ≥ 150 mg/dl triglyceride, or the use of a blood lipid-lowering medication. Chronic kidney disease (CKD) was defined as an estimated glomerular filtration rate of less than 60 ml/min/1.73 m² for ≥ 3 months.

Criteria for the clinical diagnosis of CE

CE was diagnosed based on angiographic and other diagnostic imaging findings according to a previous report [3]. The three major criteria were (1) angiographically confirmed coronary artery thrombosis without atherosclerotic components by optical frequency domain imaging (OFDI) or intravascular ultrasound (IVUS); (2) accompanying embolization in other coronary arteries; and (3) simultaneous systemic thromboembolism, excluding a thrombus in the left ventricle related to AMI. The three minor criteria consisted of (1) < 25% luminal narrowing on coronary angiography, excluding the culprit lesion; (2) evidence of an embolic source identified by any imaging modality; and (3) a complication of a potential thromboembolic pathophysiology, such as intracardiac tumor, infective endocarditis, prosthetic valve implantation, atrial fibrillation (AF), any pathology or hemostatic disease causing a hypercoagulable condition, patent foramen ovale, or atrial septal defect.

Histopathology

Aspiration thrombectomy was performed on each patient using standard PCI techniques. Thrombi were removed en bloc and fixed in 10% buffered formalin. The specimens were transected and embedded in paraffin. After hematoxylin and eosin (H&E) and elastica-Masson (EM) staining, sections of all extracted thrombi were examined. The section with the largest thrombus segment was selected for further analysis. EM is a connective tissue stain that colors elastin black and collagen and proteoglycan blue. The sections were also probed with anti-CD34 and anti-CD68 antibodies to recognize endothelial cells and macrophages in the thrombus, respectively. When calcifications were observed on HE staining, von Kossa staining was performed. All sections were evaluated by two independent observers who were blinded to the clinical findings and identities of each patient.

**Figure 1:**
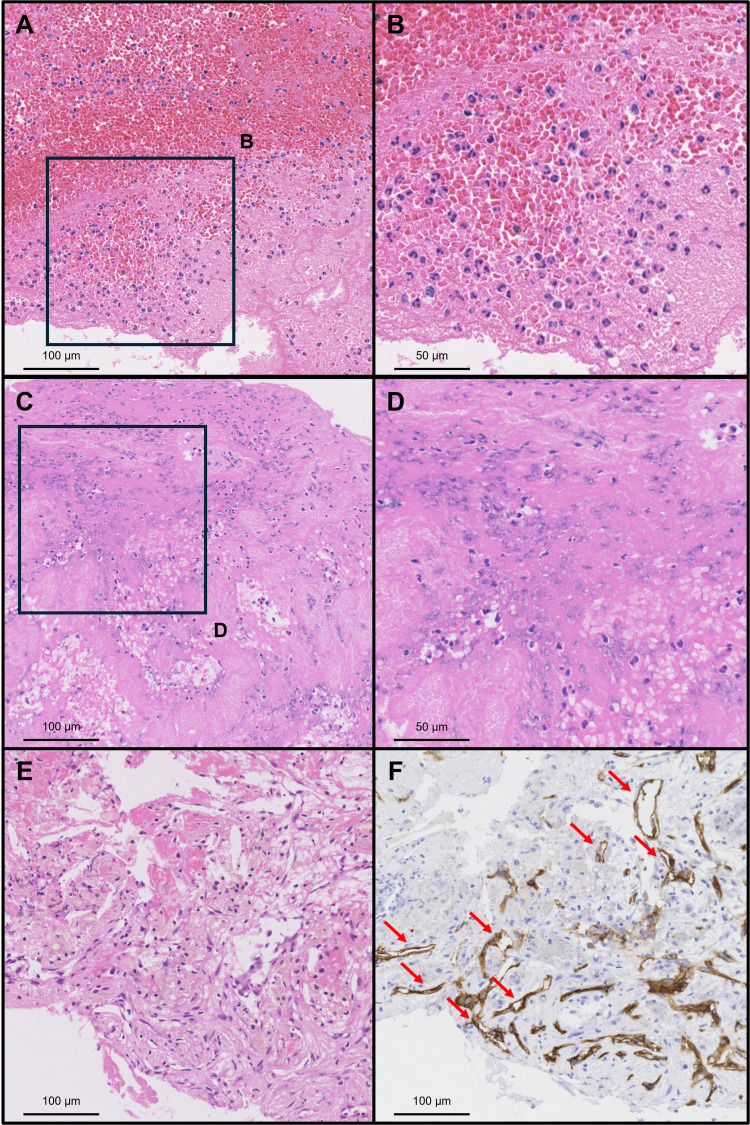
Histological images of aspirated thrombi showing thrombus age. (A, B) Fresh thrombus (< 1 day), composed of layered patterns of platelets, erythrocytes, and intact granulocytes among fibrins. (C, D) Lytic thrombus (one to five days), characterized by the colliquation necrosis and karyorrhexis of granulocytes. (E, F) Old thrombus (> 5 days) showing the ingrowth of smooth muscle cells, with or without depositions of connective tissue, capillary vessel ingrowth, and endothelialization of the surface of the thrombus (red arrows). (A–E: Hematoxylin and eosin stains; F: CD34 stain for endothelial cells; A, C, E, F: Low-power images; B, D: High-power images)

The thrombi were pathologically classified into three groups according to previously published definitions of thrombus age [19]: (1) fresh thrombus (< 1 day), composed of layered patterns of platelets, fibrin, erythrocytes, and intact granulocytes; (2) lytic thrombus (one to five days), characterized by areas of colliquation necrosis and karyorrhexis of granulocytes; and (3) old thrombus (> 5 days), which showed ingrowth of smooth muscle cells, with or without connective tissue deposition and capillary vessel ingrowth. Representative examples of fresh, lytic, and old thrombi are shown in Figure 1.

Statistical analysis

Normality of distribution was examined using the Shapiro-Wilk test. Results for continuous variables with normal distribution are expressed as mean ± SD. Variables with non-normal distributions were expressed as median (Q1-Q3). Categorical data were examined using the chi-square test or Fisher’s exact test, as appropriate. Comparisons of continuous variables with normal distributions were performed using the Student’s t-test, and the Mann-Whitney U test was used to analyze variables with non-parametric distributions. Statistical significance was set at P < 0.05. All statistical analysis was performed using JMP version 15.0 (SAS Institute Inc., Cary, NC) and Prism version 8 or 9 (GraphPad Software, La Jolla, CA).

## Results

Clinical characteristics

The study enrolled 89 patients: four with CE and 85 with non-CE. Aspirated thrombi were resected from all patients and examined histopathologically. The clinical characteristics of the two groups are shown in Table 1. The median age at the time of PCI was 66 (57-79 years) years. A higher prevalence of AF was observed in the CE group than in the non-CE group. Median HbA1c was significantly lower in the CE group than in the non-CE group (5.5% (5.3%-5.5%) vs. 5.9% (5.6%-6.6%), P = 0.015). Other baseline characteristics, including the Killip class and culprit lesions, were similar in both groups. All four cases with CE were not treated with an anticoagulant prior to admission.

**Table 1:** Clinical characteristics of the patients Values are mean ± SD, median (interquartile range), or number (%) of observations. Categorical variables are analyzed using the chi-square (χ²) test or Fisher’s exact test, as appropriate. Comparisons of continuous variables with normal distributions are performed using the Student’s t-test, and the Mann–Whitney U test is used to analyze variables with non-parametric distributions. AF: atrial fibrillation; CAD: coronary artery disease; CE: coronary artery embolism; CK: creatine kinase; CRP: C-reactive protein; eGFR: estimated glomerular filtration rate; HDL-C: high-density lipoprotein cholesterol; LAD: left anterior descending artery; LCX: left circumflex artery; LDL-C: low-density lipoprotein cholesterol; LMCA: left main coronary artery; PCI: percutaneous coronary intervention; RCA: right coronary artery; STEMI: ST-segment elevation myocardial infarction; TIMI: thrombolysis in myocardial infarction; WBC: white blood cell.

Patients	CE	Non-CE	Statistic (test)	p-value
	(n=4)	(n=85)		
Age, years	68 (56-86)	66 (57-80)	U=162	0.78
Female, n (%)	1 (25)	17 (20)	χ²=0.06	1
Body mass index, kg/m^2^	24.1 (18.6-26.8)	24.4 (21.3-26.9)	U=150	0.58
Presenting characteristics, n (%)				
Killip III or IV	0	12 (14)	χ²=0.65	1
STEMI	4 (100)	77 (91)	χ²=0.41	1
Coronary risk factors, n (%)				
Diabetes mellitus	0	33 (39)	χ²=2.5	0.29
Hypertension	1 (25)	55 (65)	χ²=2.6	0.142
Dyslipidemia	2 (50)	62 (73)	χ²=1.0	0.31
Chronic kidney disease	1 (25)	34 (40)	χ²=0.36	1
Current smoker	1 (25)	30 (35)	χ²=0.18	1
Family history of CAD	1 (25)	16 (19)	χ²=0.09	0.58
AF documentation, n (%)			χ²=31	<0.001
Paroxysmal	3 (75)	6 (7)		
Persistent	0	1 (1)		
Permanent	1 (25)	1 (1)		
No documentation	0	77 (91)		
Laboratory tests				
WBC count, /μL	7,415 (5,483-9,025)	9,610 (7,555-11,795)	U=83	0.083
Hemoglobin, g/dL	14.3±1.1	14.3±2.1	t=0.04	0.97
eGFR, mL/min/1.73m^2^	77.3 (44.7-95.5)	66.0 (49.5-82.0)	U=138	0.53
HbA1c, %	5.5 (5.3-5.5)	5.9 (5.6-6.6)	U=47	0.015
Glucose, mg/dL	132 (113-166)	160 (127-203)	U=117	0.29
LDL-C, mg/dL	90 (78-193)	123 (103-150)	U=108	0.22
Triglyceride, mg/dL	89 (47-421)	120 (83-201)	U=140	0.55
HDL-C, mg/dL	42 (37-74)	45 (39-56)	U=156	0.8
Peak CK, IU/L	2,756 (1,245-3,670)	2,306 (844-4,316)	U=174	0.9
Peak CK-MB, IU/L	222 (96-338)	257 (87-402)	U=147	0.73
CRP, mg/dL	0.13 (0.04-0.16)	0.17 (0.05-0.68)	U=110	0.24
Ejection fraction, %	55 (50-70)	52 (48-60)	U=124	0.37
Culprit lesion, n (%)			χ²=1.0	0.79
LMCA	0	1 (1)		
LAD	2 (50)	29 (34)		
LCX	0	15 (18)		
RCA	2 (50)	40 (47)		
TIMI flow grade 0-1 prior to PCI, n (%)	4 (100)	77 (91)	χ²=0.41	1
Onset to thrombectomy time, min	167 (142-273)	177 (120-321)	U=150	0.86
Medications prior to admission, n (%)				
Aspirin	0	6 (7)	χ²=0.30	1
P2Y12 inhibitor	0	3 (4)	χ²=0.15	1
Statins	0	11 (13)	χ²=0.59	1
CHA₂DS₂-VASc score	2 (0-4)	2 (1-3)	U=144	0.59
HAS-BLED score	1 (0-2)	1 (1-2)	U=130	0.39

Histopathological characteristics of the aspirated thrombi

**Figure 2:**
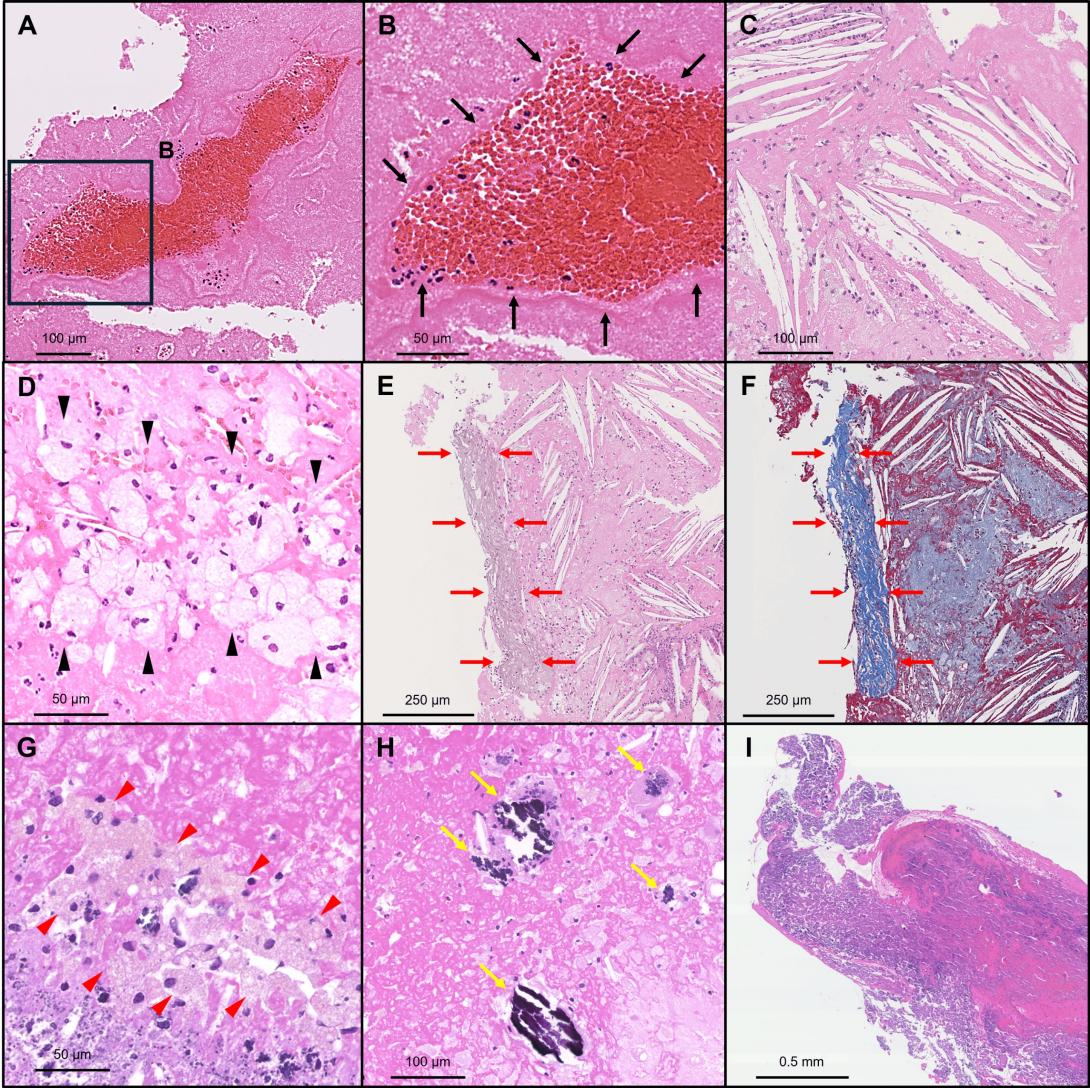
Representative coronary thrombus images for pathological analyses (A, B) Erythrocytes (black arrows) surrounded by fibrin. (C) Cholesterol crystals are present in needle-shaped form. (D) The accumulation of foamy macrophages (black arrowheads). (E, F) Collagen tissue (red arrows) with cholesterol crystals. (G) The accumulation of hemosiderin-laden macrophages (red arrowheads) with light-brown pigments inside the cells around erythrocytes and fibrin. (H) Fragmented calcification (yellow arrows). (I) Nodular calcification, including calcification with fibrin. (A–E, G–I: Hematoxylin and eosin stains; F: Elastica-Masson stain; A, C, E, F, H, I: Low-power images; B, D, G: High-power images)

Various histopathological characteristics of the aspirated samples are shown in Table 2. The representative pathological parameters of the aspirated thrombi are shown in Figure 2. Median erythrocytes and erythrocytes/total thrombus areas ratio were significantly greater in the CE group when compared to the non-CE group (4.94 mm² (3.38-7.35 mm²) vs. 0.62 mm² (0.05-2.51 mm²), P = 0.011; 55.6% (50.9%-57.7%) vs. 12.6% (2.0%-36.2%), P = 0.002). The prevalence of erythrocyte-dominant thrombi was significantly higher in the CE group than in the non-CE group (75% vs. 7%; P = 0.003).

**Table 2:** Histopathological characteristics of aspirated thrombi Values are median (interquartile range) or number (%) of observations. Categorical data are examined using the chi-square test or Fisher’s exact test, as appropriate. The Mann–Whitney U test is used to analyze variables with non-parametric distributions. CE: coronary artery embolism; TTA: total thrombus area

Patients	CE	Non-CE	Statistic (test)	p- value
	(n=4)	(n=85)		
TTA, mm^2^	9.07 (6.34-13.0)	3.82 (1.75-7.57)	U=77	0.066
Area of erythrocytes, mm^2^	4.94 (3.38-7.35)	0.62 (0.05-2.51)	U=41	0.011
Area of erythrocytes/TTA, %	55.6 (50.9-57.7)	12.6 (2.0-36.2)	U=17	0.002
Erythrocyte-dominant thrombi, n (%)	3 (75)	6 (7)	χ²=19	0.003
Age of aspirated thrombi, n (%)			χ²=8.4	0.015
Fresh	3 (75)	15 (18)		
Lytic	1 (25)	24 (28)		
Old	0	46 (54)		
Any plaque components, n (%)	0	44 (52)	χ²=4.1	0.117
Cholesterol crystals, n (%)	0	34 (40)	χ²=2.6	0.29
Foamy macrophage, n (%)	0	37 (43)	χ²=3.0	0.138
Hemosiderin-laden macrophage, n (%)	0	29 (34)	χ²=2.0	0.3
Collagen tissue, n (%)	0	20 (24)	χ²=1.2	0.57
Calcification, n (%)	0	15 (18)	χ²=0.85	1

**Figure 3:**
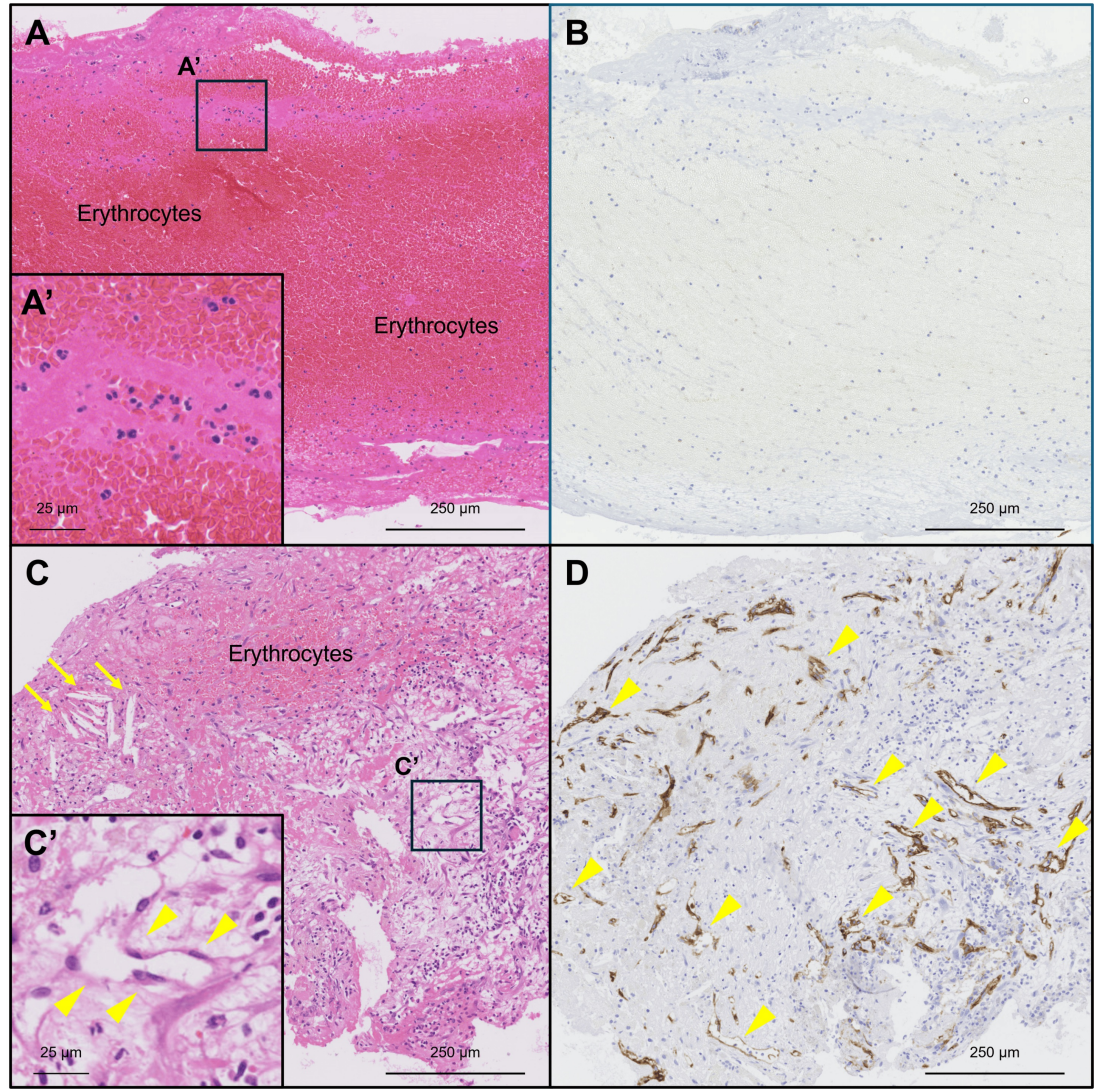
The representative aspirated thrombi derived from patients with (A, B) CE and (C, D) non-CE (A, B) A microphotograph of the thrombus from a 79-year-old man with AMI due to CE shows erythrocyte-dominance and intact granulocytes (A’) and no capillary vessel ingrowth (B), which suggests a ‘fresh’ thrombus. (C, D) A microphotograph of the thrombus from a 75-year-old man with AMI due to non-CE shows a comparatively small area of erythrocytes and capillary vessel ingrowth (yellow arrowheads in C’ and D), which indicates an ‘old’ thrombus. Cholesterol crystals are also observed (yellow arrows). AMI = acute myocardial infarction CE: coronary artery embolism. (A, C: Hematoxylin and eosin stains; B, D: CD34 stain; A–D: Low-power images; A’, C’: High-power images in A and C, respectively)

Among 89 patients with de novo AMI, 18 (20%) had “fresh” thrombi, 25 (28%) had “lytic” thrombi, and 46 (52%) had “old” thrombi. The age of aspirated thrombi was significantly fresher in the CE group compared to the non-CE group (“fresh”: 75% vs. 18%; “lytic”: 25% vs. 28%; and “old”: 0% vs. 54%, respectively). Plaque components, including cholesterol crystals, collagen tissue, and calcification, were not present in the specimen from the CE group. Among the four cases of CE, two paroxysmal and one permanent patient with AF had “fresh” thrombi, whereas one patient with paroxysmal AF had “lytic” thrombi. Representative aspirated thrombi derived from the CE and non-CE groups are shown in Figure 3.

## Discussion

The major findings of this study were 1) thrombi aspirated from patients with AMI due to CE contained significantly more erythrocytes than those aspirated from patients with AMI due to atherosclerotic causes, and 2) aspirated materials from patients with AMI due to CE included fresher thrombi than those from atherosclerotic causes. To the best of our knowledge, this study is the first to perform a pathological analysis of aspirated coronary thrombi from patients with CE by directly comparing them with non-CE patients undergoing aspiration thrombectomy during PCI for AMI.

Comparison with previous studies

The prevalence of CE in patients with AMI has been reported to be 3%-13% based on diagnostic imaging and autopsy studies [20-22]. In our study, four out of 99 patients with AMI (4%) were diagnosed with CE after applying the previously reported diagnostic criteria [3]. Regarding etiology, the three main causes of CE were reported to be AF (73%), followed by malignancy (10%), and cardiomyopathy (9%) [3]. Conversely, the etiology of each CE case was attributed to AF. In a study of 1,232 consecutive patients presenting with de novo AMI, the CE and non-CE groups had similar age and coronary risk factors, except for body mass index and smoking (23.9 (22.9-24.1) vs. 26.8 (24.2-30.0), P < 0.001; 43.4% vs. 58.9%, P = 0.025, respectively) [21]. In this study, age, sex, and coronary risk factors were not significantly different in the CE group except for HbA1c, compared with the non-CE group (P = 0.015, Table 1), which suggests that dysglycemia is associated with AMI due to atherosclerotic cause, compared to AMI due to CE. Our results are consistent with those of previous studies.

Erythrocyte-dominant thrombi in CE

Generally, thrombi of arterial atherosclerotic origin are understood to be platelet-rich, while those of cardiac origin, including AF, are erythrocyte/fibrin-rich [23,24]. From the multi-institutional Stroke Thromboembolism Registry of Imaging and Pathology (STRIP registry), Fitzgerald et al. reported that the prevalence of platelet-rich clots (55% vs. 21%, P = 0.005) and the ratio of platelet component (22 ± 4% vs. 14 ± 14%, P = 0.03) were significantly higher in the atherosclerotic-origin group compared to the cardiac group [23]. In a histopathological analysis of retrieved thrombi from 37 patients with acute stroke, Kim et al. found that thrombi of cardiac origin contained a significantly higher percentage of erythrocytes (38% vs. 17%, P = 0.031) and a lower percentage of fibrin (33% vs. 49%, P = 0.044) than those from large-artery atherosclerosis [24]. A pathological study using scanning electron microscopy showed that the continuation of AF can lead to the creation of endothelial craters, resulting in the formation of a thrombotic mass consisting mainly of erythrocytes at the location of severe endothelial damage [25]. This may explain the erythrocyte-dominant thrombi observed in patients with AMI due to CE in this study (Table 2).

Previous studies have shown that erythrocyte-rich thrombi aspirated by thrombectomy are associated with poor prognosis in patients with AMI [14,16,18]. Yunoki et al., using aspirated thrombi from 178 patients with STEMI, reported that the incidence of distal embolization during PCI was higher in the erythrocyte-rich group (P < 0.001), and the occurrence of lower TIMI grade flow and progression of left ventricular remodeling six months after PCI were higher in the erythrocyte-rich group than in the non-erythrocyte-rich group (P < 0.01, P < 0.01, respectively) [14]. Yang et al. found that in-hospital major adverse cardiovascular events were more frequently observed in the red thrombus (erythrocyte-rich thrombus) group than in the white thrombus (platelet-rich thrombus) group in a pathological analysis of aspirated specimens from 97 patients with AMI. (15.6% vs. 0%) [18]. Recent studies have shown that erythrocytes play an important role in thrombus formation. Cines et al. demonstrated that erythrocytes in the thrombus could change shape from biconcave to polyhedral, leading to tight clot packing and resistance to fibrinolysis [26]. This may partially explain the poor prognosis of AMI patients with CE.

Fresher aspirated thrombi from CE than those from an atherosclerotic cause

A previous investigation reported that thrombus formation in the left atrial appendage occurs within three days after the continuation of AF [27]. In a pathological analysis of stroke thrombi retrieved via mechanical thrombectomy, Koge et al. reported that 63% (106/168) of patients with ischemic stroke complicated by AF presented with fresh thrombi [28], which is consistent with our finding that fresh thrombi were observed in 75% of patients with CE (Table 2). In contrast, lesion destabilization frequently occurs in unstable atherosclerotic coronary plaques, days or weeks before the onset of AMI [19]. These findings support our observation that aspirated thrombi from CE were fresher than those from non-CE (atherosclerotic cause) (Table 2).

A paucity of data exists regarding the direct pathological comparison of aspirated thrombi between patients with AMI. Kotooka et al. reported three cases of AMI due to CE [29] and showed that all three patients had fresh thrombi in the pathological analysis of aspirated thrombi. Despite the limited number of patients, we demonstrated for the first time that aspirated thrombi in the coronary artery were fresher in patients with AMI due to CE than in those with AMI due to atherosclerosis.

Clinical implications

Identifying the etiology of AMI is crucial for secondary prevention. Owing to current advancements in technology, the etiology of AMI has been identified using imaging modalities such as optical coherence tomography (OCT), OFDI, and IVUS. However, this can be challenging because plaque rupture is not always clearly seen on IVUS due to poor imaging resolution, and OCT or OFDI may fail to visualize culprit lesions when massive thrombus or wire artifacts are present. The histological characteristics of aspirated thrombi in patients with AMI may offer valuable clues to their etiology, thereby contributing to decision-making regarding secondary prevention strategies. Aspiration thrombectomy followed by anticoagulation therapy instead of stent implantation may be an optimal and effective strategy when the underlying pathophysiological mechanism of AMI is CE.

Limitations of our study

The sample size of this cross-sectional study, conducted at a single center, was small. The CE group had only four patients. These results need to be confirmed in a larger, multicenter study. Second, aspiration thrombectomy can be affected by the operator’s procedure during PCI, and the composition of aspiration thrombi does not always reflect that of the entire thrombus in the culprit lesions. Third, we did not perform a landmark analysis for coronary thrombotic events using Kaplan-Meier curves between patients with and without anticoagulant therapy because of the small sample size in this study. 

## Conclusions

This cross-sectional pathological analysis of aspirated coronary thrombi suggests that aspirated thrombi derived from patients with AMI due to CE are more erythrocyte-dominant and fresher than those from AMI due to atherosclerotic causes. Further pathological studies on a large number of cases are needed to confirm these findings and to speculate on the recommendations for secondary prevention, including antithrombotic therapy, after AMI.
